# Impact of Phosphatic Nutrition on Growth Parameters and Artemisinin Production in *Artemisia annua* Plants Inoculated or Not with *Funneliformis mosseae*

**DOI:** 10.3390/life12040497

**Published:** 2022-03-29

**Authors:** Valeria Todeschini, Flavio Anastasia, Nadia Massa, Francesco Marsano, Patrizia Cesaro, Elisa Bona, Elisa Gamalero, Ludovica Oddi, Guido Lingua

**Affiliations:** 1 Dipartimento di Scienze ed Innovazione Tecnologica, Università del Piemonte Orientale, 15121 Alessandria, Italy; flavio.anastasia@uniupo.it (F.A.); nadia.massa@uniupo.it (N.M.); francesco.marsano@uniupo.it (F.M.); patrizia.cesaro@uniupo.it (P.C.); elisa.gamalero@uniupo.it (E.G.); guido.lingua@uniupo.it (G.L.); 2 Dipartimento per lo Sviluppo Sostenibile e la Transizione Ecologica, Università del Piemonte Orientale, 13100 Vercelli, Italy; elisa.bona@uniupo.it; 3 Dipartimento di Scienze della Vita e Biologia dei Sistemi, Università degli Studi di Torino, 10123 Torino, Italy; ludovica.oddi@unito.it

**Keywords:** mugwort, P supply, AM fungi, secondary metabolites

## Abstract

*Artemisia annua* L. is a medicinal plant appreciated for the production of artemisinin, a molecule used for malaria treatment. However, the natural concentration of artemisinin *in planta* is low. Plant nutrition, in particular phosphorus, and arbuscular mycorrhizal (AM) fungi can affect both plant biomass and secondary metabolite production. In this work, *A. annua* plants were ino- culated or not with the AM fungus *Funneliformis mosseae* BEG12 and cultivated for 2 months in controlled conditions at three different phosphatic (P) concentrations (32, 96, and 288 µM). Plant growth parameters, leaf photosynthetic pigment concentrations, artemisinin production, and mineral uptake were evaluated. The different P levels significantly affected the plant shoot growth, AM fungal colonization, and mineral acquisition. High P levels negatively influenced mycorrhizal colonization. The artemisinin concentration was inversely correlated to the P level in the substrate. The fungus mainly affected root growth and nutrient uptake and significantly lowered leaf artemisinin concentration. In conclusion, P nutrition can influence plant biomass production and the lowest phosphate level led to the highest artemisinin concentration, irrespective of the plant mineral uptake. Plant responses to AM fungi can be modulated by cost–benefit ratios of the mutualistic exchange between the partners and soil nutrient availability.

## 1. Introduction

*Artemisia annua* L. is an herbaceous, annual aromatic plant belonging to the Aste-raceae family [1,2], native of Asia, and widely distributed in temperate and subtropical areas of the world [3]. It is well adapted to different types of soils, and it has no particular nutritional requirements. However, its growth is stimulated by phosphorus and potassium supply, even if in small quantities [4,5]. This plant is appreciated for the production of artemisinin, a molecule used for the treatment of malaria [6], one of the biggest health problems in developing countries (World Health Organization—WHO) [7]. Indeed, the WHO recommends the use of the artemisinin-based combination therapies as the best treatment currently available against malaria [2,7,8,9]. Artemisinin is a sesquiterpene lactone produced in the biseriate glandular trichomes of artemisia leaves [10,11,12,13]. The synthesis of this molecule is based on the existence of two distinct pathways: (i) the cytosolic mevalonate (MVA) pathway and (ii) the plastidial methyl erythritol phosphate (MEP) pathway [10,14,15,16]. Recent studies have highlighted that artemisinin is mainly synthesized from the linear isoprene precursors farnesyl pyrophosphate (FPP), whose biosynthesis is due to a cross talk between the above-mentioned biosynthetic pathways [17].

The market demand for artemisinin and its derived compounds is large, but their production is expensive from an economic point of view. Moreover, the biosynthesis of this molecule implies an energy cost for plants. Plant genotype, environmental and growth conditions, and agronomic practices, together with the low concentration of plant isoprenoids (1–15% of plant dry weight (DW)), are important factors to be considered for artemisinin production and commercialization [18,19]. The artemisinin content in *A. annua* plants is low (0.01–1% DW) [20]. To promote plant growth and then enhance artemi-sinin concentration, different approaches (molecular, physiological, and biochemical) have been explored [21,22,23,24,25,26,27,28]. Unfortunately, biotechnological methods (in vitro hairy root cultivation, plant cell cultures, and fermentation with microbes) have not been found to be significantly effective [29,30]. For this reason, strategies leading to an increase in artemisinin concentration and plant yield in cultivated plants represent an important research field.

Many studies have highlighted that arbuscular mycorrhizal (AM) fungi can also modulate secondary metabolite and biomass production in *A. annua* [21,22,24,28,31]. In fact, it is well known that the symbiosis between the plant root system and AM fungi can improve plant growth, physiology, metabolism, and fitness [32,33,34,35,36,37,38]. AM fungi belonging to the phylum Glomeromycota [39] are obligate biotrophs, whose life cycle depends on the host plant. The symbiosis is mutualistic: while the plant provides photosynthates to the fungus, the fungus improves plant water uptake and mineral nutrition, especially the phosphatic (P) one [32,40,41,42,43,44]. This bidirectional exchange occurs in the arbuscule, the active site of the symbiosis [45,46,47]. The ability of AM fungi to colonize plant roots not only depends on the specificity of plant–fungus interactions but also is strongly affected by the phosphorus concentration in the soil. Many studies have reported that the phosphate concentration in the soil significantly affects the ability of AM fungi to colonize plant roots [48]. In turn, both factors (phosphate availability and AM symbiosis) can influence plant growth, mineral uptake, photosynthetic pigment concentration, and plant secondary metabolite production [11,28,34,35,38,44,49,50,51,52]. Nevertheless, some works have reported neutral or negative effects of AM fungi on plant fitness [42,53,54,55,56,57,58,59,60].

Phosphorus is an essential nutrient for the growth and productivity of plants. On the one hand, it plays a crucial role in different biological processes, such as ATP production, nucleic acid (DNA and RNA) synthesis, photosynthesis, glycolysis, respiration, membrane synthesis and stability, activation/inactivation of various enzymes, redox reactions, cellular signaling, carbohydrate metabolism, and nitrogen uptake [61,62,63,64]. On the other hand, due to its low bioavailability in the soil, phosphorus is often a limiting element for plant growth [62,65]. Moreover, the presence of phosphorus in soil (or in the growth substrate) could affect the availability of other elements. As an example, phosphorus interaction with both macro- (especially N and K) and micro- (mainly Cu, Zn, Fe, Mo, and B) nutrients in different crops could be either synergistic or antagonistic [64]. Many studies have shown an increase in plant biomass in relation to a high concentration of bioavailable phosphate in the growth medium ([64] and references therein), but only few works refer to *A. annua* plants [11,21,43,64]. The secondary plant metabolism may also be affected by the phosphate concentration and bioavailability in the growth substrate [11,21,66,67]. However, relatively few studies have reported an increase in the artemisinin content in *A. annua* plants in the presence of high bioavailable phosphate levels [11,21,22].

*Funneliformis mosseae* BEG 12 is one of the most-used AM fungal species able to e-stablish symbiosis with many taxonomically distant plants. However, in the literature, only a few studies regarding the effects of this fungus (applied as pure culture) on >*A. annua* plants [24,31] grown under controlled conditions (growth chamber) with different P nutrition levels have been reported. Therefore, in this work, we aim to assess the effects of three different levels of P nutrition on mycorrhizal and non-mycorrhizal *A. annua* plants in terms of plant growth parameters, photosynthetic pigment concentration, leaf artemi-sinin production, and mineral uptake.

## 2. Materials and Methods

### 2.1. Mycorrhizal Inoculum

The inoculum of the AM fungus *F. mosseae* (BEG12, European Bank of Glomales, Dijon, France) was propagated in a pot, starting from surface-sterilized spores inoculating pre-germinated sorghum (*Sorghum bicolor* L.) seeds, grown in sterile quartz sand for 4 months in controlled conditions (a 16 h/8 h light/dark photoperiod at a light intensity of 150 µmol m^−2^ s^−1^). The obtained inoculum was formed by a mixture of quartz sand, fragments of sorghum roots, fungal hyphae, and spores.

### 2.2. Experimental Design

*A. annua* seeds (var. Anamed) were sterilized in a sodium hypochlorite solution (5%, *v*/*v*) for 5 min, washed three times (5 min each), and soaked for 1 h in deionized sterile water. Sowing was carried out in 500 mL capacity pots: at the bottom of the pots, a layer of sterile quartz sand (size 4/5 mm) was used to ensure optimal drainage. The growth substrate consisted of peat and quartz sand of varying granulometry. The peat (K Select, Klasmann–Deilmann; pH 6.0) was previously sterilized by flowing steam (104 °C for 1 h). After steam sterilization, the peat vessels were put in an oven at 40 °C for 6 h to reduce humidity and to avoid mold formation. The entire quartz sand was sterilized in an oven at 180 °C for 4 h. The plants were inoculated (five plants per treatment) or not (seven plants per treatment) with the AM fungus *F. mosseae* BEG12 after mixing the inoculum (80 mL/plant corresponding to about 7200 propagules) with the sterile growth medium. After sowing, the pots with the seeds were irrigated with 200 mL of sterile deionized water and left in a growth chamber with a 16 h/8 h light/dark photoperiod at a light intensity of 150 µmol m^−2^ s^−1^. The temperature was 25 °C in the light and 21 °C in the dark. The plants were irrigated three times a week with a Long Ashton nutrient solution at three different P concentrations (32 µM, 96 µM, and 288 µM) (Table 1) and harvested after 60 days of growth in controlled conditions. The Long Ashton solution consisted of 5 macronutrient solutions (Ca(NO_3_)_2_ *×* 4H_2_O (2 mM), MgSO_4_ × 7H_2_O (0.75 mM), KNO_3_ (2 mM), FeNa EDTA (50 µM), and NaH_2_PO_4_ (32 µM)) and a micronutrient solution (MnSO_4_ × H_2_O (10 µM), CuSO_4_ × 5H_2_O (1 µM), H_3_BO_3_ (40 µM), ZnSO_4_ × 7H_2_O (2 µM), NaCl (100 µM), and Na_2_MoO_4_ × 2H_2_O (0.5 µM)). All solutions were sterilized at 121 °C for 15 min.

### 2.3. Mycorrhizal Colonization in the Root System

Sixty randomly chosen 1 cm long root pieces from each plant were clarified in 10% KOH in a water bath at 60 °C for 20 min. Then, the samples were stained with 1% lactic blue (methyl blue 1% in lactic acid). The excess dye was removed with a series of lactic acid washes. Finally, the samples were stored at 4 °C for 24 h in lactic acid. The following day, the obtained samples were mounted on a slide and observed under an optical microscope: two slides for each plant were prepared (30 root pieces for each slide). Typical AM fungal structures (hyphae and arbuscules) inside *A. annua* root are shown in Figure S1. According to Trouvelot et al. [68], each root piece was attributed to a class based on the mycorrhizal presence. Then, the frequency of mycorrhization (F%), the percentage of co-lonized root tissue (M%), and the abundance of arbuscules (A%) were calculated.

### 2.4. Plant Parameters

For each plant, stem height, root length, and shoot, root, and leaf fresh weights (FWs) (weighted with an analytical-grade balance) were recorded at harvest; then, shoot, root, and leaf samples were dried at 60 °C for 1 week in an oven for dry weight (DW) determination.

### 2.5. Analysis of Leaf Photosynthetic Pigments

Chlorophyll a and b and carotenoid concentrations were determined according to Porra et al. [69] using a 0.1–0.5 nm resolution range spectrophotometer. Briefly, 0.02 g of fresh leaves from each plant were kept in the dark at 4 °C in N, N-dimethylformamide (1.5 mL) for a week, i.e., until complete pigment extraction. The concentrations of chlorophylls and carotenoids were evaluated spectrophotometrically using the following wavelengths: 663.8 nm, 646.8 nm, and 480 nm.

### 2.6. Leaf Extraction, HPLC Analysis, and MS Detection of Artemisinin

The dried leaves were finely crushed by mortar and pestle to obtain a homogenous compound, which was used for artemisinin extraction according to Lapkin et al. [70], with some modifications. Briefly, for each extraction, 12.5 mL of acetone 100% were added to 0.5 g of leaf dry material (of each plant) in a centrifuge tube. The sample was stirred at 250 rpm for 30 min at room temperature and centrifuged at 13,000 rpm for 60 min at 22 °C. The supernatant was filtered with 0.22 µm filter, concentrated in a Thermo Scientific Savant SpeedVac vacuum concentrator for 30 min, resuspended in 900 µL of mobile phase (50% acetonitrile, 30% HPLC water, and 20% methanol), and left to precipitate for 1 h at room temperature. Then, it was centrifuged at 13,000 rpm and 20 µL of each sample was diluted 1:10 in the mobile phase, filtered with a 0.22 µm filter, loaded into vials, and analyzed with HPLC. The used HPLC system (Dionex, Sunnyvale, CA, USA) consisted of a solvent delivery module (Ultimate 3000 pump LPG-3400A), an integrated vacuum degassing system and a mixing chamber, a UV detection module (UVD-3000), and an autosampler (WPS-3000TSL Analytical). The system was controlled by Chromeleon software (version 6.70 SP7), which is also used for data processing. Artemisinin detection was performed at 215 nm, using an injection volume of 20 µL. The artemisinin calibration curve was constructed using different concentrations (0.5, 1, and 2 mg mL^−^^1^) of an analytical standard (artemisinin No. 69532—10 mg, Sigma-Aldrich, Darmstadt, Germany). The chromatographic run was performed in isocratic mode with a mobile phase consisting of acetonitrile, HPLC water, and methanol (5:3:2; *v*/*v*/*v*) at a flow rate of 1 mL/min and a temperature of 45 °C. In addition, the artemisinin peak was identified in comparison to the retention time of the artemisinin standard and through the injection of the sample containing a “spike” of the analytical standard. All chemicals were analytical-grade reagents (Sigma-Aldrich, Darmstadt, Germany). An example of a chromatogram obtained from C and M plants grown at 32 µM of phosphate is reported in the supplemental materials (Figure S2A). The artemisinin was also detected with mass spectrometry: the artemisinin peak obtained by HPLC analysis was collected, concentrated, and analyzed by direct injection into a MALDI-TOF analysis Voyager DE-PRO mass spectrometer (AB-Sciex, Concord, ON, Canada) equipped with a nano-electrospray source of positive ionization applying a voltage of 1600 V. An MS spectrum of protonated artemisinin was reported in supplemental materials (Figure S2B).

### 2.7. Element Concentrations by ICP-OES Analysis

The dried shoot and root samples of each plant were finely powdered for ICP–OES analysis and digested following the method of Ene-Obong et al. [71] with some modifications, i.e., 5 mL of 65% nitric acid (HNO_3_) were added to 0.1 g of dried material. The obtained samples were digested in a CEM MARS microwave (CEM corporation, Cologno al Serio, BG, Italy). For the element determination, an inductively coupled plasma with optical emission (ICP-OES, Spectro Genesis, Ametek—Berwyn, PA, USA) equipped with a cross-flow-type nebulizer and a Scott double-pass nebulization chamber was used. The samples were introduced into the nebulizer system via an ASX-260 autosampler (CETAC Technologies—Omaha, NE, USA). Argon plasma was generated inside a 1.8 mm injector torch at 1400 W. The cooling, auxiliary, and nebulizing gas flows were set at 14.00 mL min^−^^1^, 0.80 mL min^−^^1^, and 0.96 mL min^−^^1^, respectively. The peristaltic pump that handled the sample introduction and discharge was set at speed 2 on a scale of 1 to 5, except for the pre-wash phase, for which it worked at speed 4. The signals recorded by the instrument were processed by the software Smart Analyzer Vision, version 5.06, and refer to the average of three detections of the same solution. Quantitative analysis was performed after calibrating the instrument with multi-element standard solutions diluted in a 1% HNO_3_ solution in ultrapure water. The same solution was used for the dilution of the mineralized samples (1:10 dilutions for P, Na, Mg, Al, Mn, and Fe and 1:100 for K and Ca) and also kept as blank. Each element was quantified considering the following emission spectral lines: P 177.495 nm, Na 589.592 nm, Mg 285.213 nm, S 180.731 nm, and K 766.491 nm. The spectral line of Ar at 404.442 nm was also checked to verify the accuracy of the analysis.

### 2.8. Statistical Analysis

The mean value and the relative standard error of the considered parameters were calculated. The obtained data were compared by means of the ANOVA test followed by the Fisher’s post-hoc comparison test. Differences among the treatments were considered significant for *p*-values < 0.05 and highly significant for *p*-values < 0.001. Furthermore, data were analyzed by a two-way ANOVA using phosphatic nutrition (P) and Fungus (F) as factors. Processing was carried out using Statview 4.5 software (Abacus Concepts).

The data were also used for multivariate statistical tools, such as principal component analysis (PCA), using the software R ver. 3.0.2. The analyzed parameters were labeled with the following acronyms, reported in brackets: frequency of mycorrhization (F), percentage of colonized root tissue (M), abundance of arbuscules (A), shoot fresh weight (FWS), shoot dry weight (DWS), stem height (HS), leaf dry weight (LW), root fresh weight (FWR), root dry weight (DWR), root length (HR), ratio between root and shoot dry weight (RSDW), shoot ratio of dry and fresh weight (DWFWS), root ratio of dry and fresh weight (DWFWR), leaf chlorophyll a (Ca) and b (Cb) concentration, ratio of leaf chlorophyll a and b concentration (CaCb), leaf carotenoid concentration (Cxc), and leaf artemisinin concentration (Artem). Data were normalized and processed with the “princomp” command; a biplot related to the scores and loadings was produced.

## 3. Results

### 3.1. Mycorrhizal Colonization

**Figure 1:**
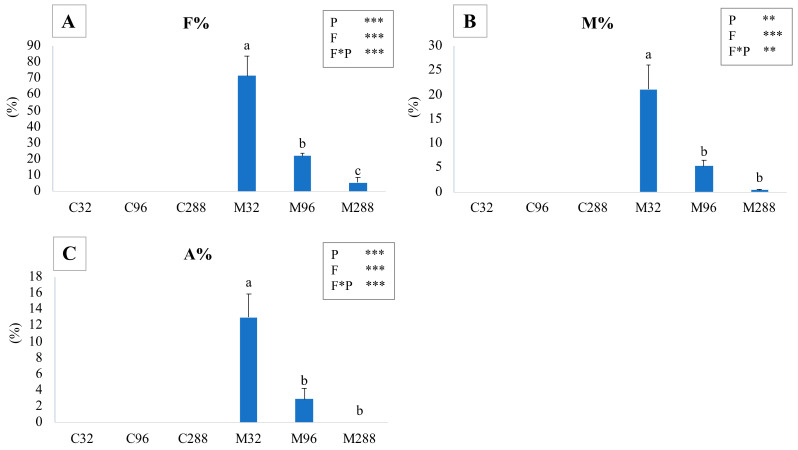
Mean values and relative standard errors of the colonization frequency F% (**A**), colonization intensity percentage M% (**B**), and percentage of arbuscule abundance A% (**C**) in *A. annua* plants grown at different P levels. C32, C96, and C288: uninoculated plants grown at 32, 96, and 288 µM of phosphate, respectively; M32, M96, and M288: plants inoculated with *F. mosseae* and grown at 32, 96, and 288 µM of phosphate, respectively. Different letters indicate significant differences between the various treatments. A two-way ANOVA is present in the inset of each graph and considers the two factors phosphatic nutrition (P) and fungus (F) and their interaction (F * P). * *p* < 0.05; ** *p* < 0.01; *** *p* < 0.0001.

Uninoculated control plants did not show any trace of mycorrhizal colonization (Figure 1). Establishment of the symbiosis was impaired by an increase in the P concentration in the growth substrate. In fact, the highest values of these parameters (frequency of mycorrhization (F%), percentage of colonized root tissue (M%), and abundance of arbuscules (A%)) were observed in M32 plants (70%, 20%, and 13%, respectively) if compared to both M96 and M288 plants. The two-way ANOVA highlighted that fungus (F), phosphate (P), and their interaction (F * P) significantly affected the considered parameters.

### 3.2. Biomass Production

In general, the increase in P concentration in the growth medium improved the epigeous biomass production both in C and M plants (Table 2). An opposite trend was observed for the root biomass (length and weight) in uninoculated plants, while no significant differences were recorded in M plants whatever the P level. The stem height reached the highest values in C96 plants, which were different from all the other treatments (ino-culated or not) except for C288 plants.

The shoot DW/FW ratio (Table 2) showed a different trend in control and inoculated plants. No differences were recorded between M plants except for M288 ones (which showed the lowest values compared to all the other treatments), while a significant decrease was observed in C plants at increasing P concentration. C32 plants presented the highest values of shoot DW/FW ratio with significant differences if compared to all the other treatments.

The highest value of DW/FW root ratio (Table 2) was observed in M32 plants, which were similar to M96 ones, while it differed from all the other treatments. Moreover, fungal inoculation affected this parameter (as shown by the two-way ANOVA; Table 2) and significant differences were observed between C and M plants grown at the same P level except for those grown at 288 µM of phosphate.

The ratio between root and shoot FW (or DW) (Table 2) strongly changed according to the different P levels, as shown by the two-way ANOVA, where the P factor significantly influenced the analysis. In general, these parameters decreased with an increasing P concentration in the substrate. This was more evident in controls, where C32 plants showed significant differences if compared to all other treatments. On the contrary, no differences were detected between M plants. Mycorrhizal inoculation (F factor) did not affect the root/shoot DW ratio, while it was the main factor (together with P and F * P) leading to the different values recorded in the root/shoot FW ratio.

The stem height/root length ratio (Table 2) showed a trend similar to that reported before for the epigeous biomass production, increasing proportionally to the enhancement of P concentration in C plants. Accordingly, the two-way ANOVA showed that the P factor was responsible of this trend. Independently of the various P levels in the substrate, mycorrhizal plants did not show any significant differences. The factor “fungus” did not affect the considered parameter. Moreover, C and M plants grown at the same P level presented similar values except for those grown at 32 µM of phosphate.

Generally, the two-way ANOVA (Table 2) revealed that P nutrition had more influence (*p* < 0.01; *p* < 0.0001) on the aboveground part of the plant if compared to the F factor (*p* < 0.05; ns). On the contrary, fungal inoculation (F) mainly influenced the root growth. The interaction between the two factors (F * P) also affected the growth parameters, al-though to a lesser extent.

### 3.3. Photosynthetic Pigment Concentration

**Figure 2:**
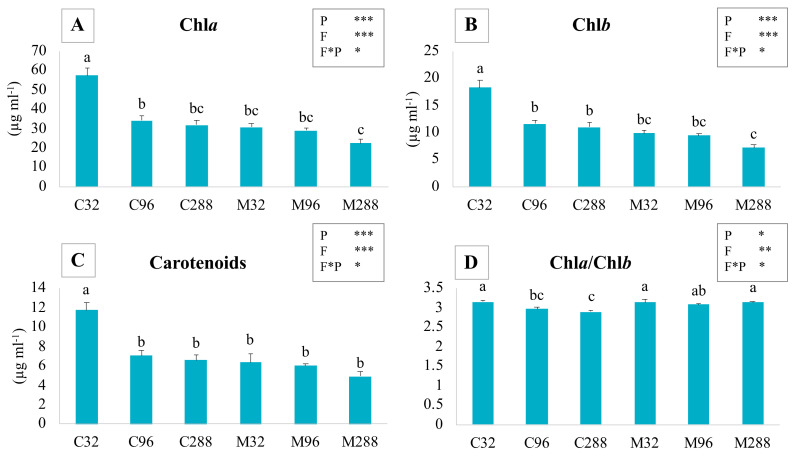
The graphs report the mean values and the relative standard errors of chlorophyll *a* (Chl*a*—(**A**)), chlorophyll *b* (Chl*b*—(**B**)), and carotenoid concentration (**C**), besides the Chl*a*/Chl*b* ratio (**D**) in the leaves of *A. annua* plants grown at different P levels. C32, C96, and C288: uninoculated plants grown at 32, 96, and 288 µM of phosphate, respectively; M32, M96, and M288: plants inoculated with *F. mosseae* and grown at 32, 96, and 288 µM of phosphate, respectively. Different letters indicate significant differences between the various treatments. Moreover, a two-way ANOVA is presented in the inset of each graph and considers the two factors phosphatic nutrition (P) and fungus (F) and their interaction (F*P): ns not significant; * *p* < 0.05; ** *p* < 0.01; *** *p* < 0.0001.

A significant decrease in the concentration of photosynthetic pigments (chlorophyll a (Chla), chlorophyll b (Chlb), and carotenoids) (Figure 2A–C) was observed in all treatments if compared to C32 plants, which showed the highest values. The enhancement of P concentration in the substrate was associated to a decreasing concentration of pigments in leaves, although the differences were not always significant. The P factor was responsible for this trend, as confirmed by the two-way ANOVA. The concentration of photosynthetic pigments in M plants was lower than that recorded in C plants grown at the same P level, with significant differences between C32 and M32 plants. In this latter case, inoculation with AM fungi negatively affected these parameters.

The Chla/Chlb ratio (Figure 2D) showed two different trends in C and M plants. While in the control plants, this ratio was inversely correlated to the P concentration in the substrate, in mycorrhizal ones, this correlation did not occur. C288 plants showed the lowest value of the Chla/Chlb ratio, with significant differences either from inoculated plants grown at the same P level or from both C32 and M32 plants. A two-way ANOVA underlined the influence of the two factors F (in a more marked way) and P, besides their interaction.

### 3.4. Artemisinin Concentration in Leaves

**Figure 3:**
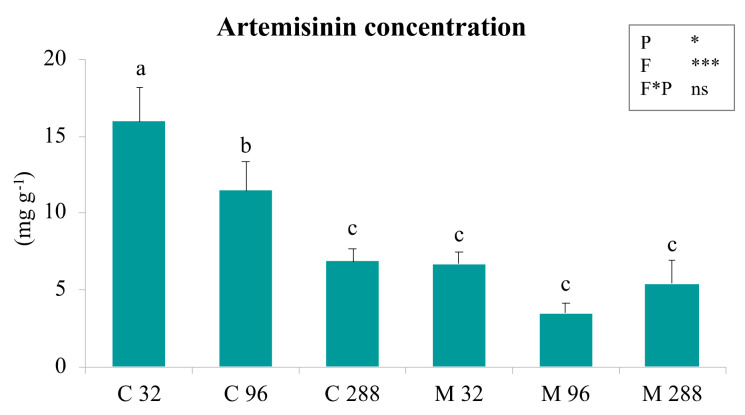
The mean values and the relative standard errors of leaf artemisinin concentration in *A. annua* plants grown at different P levels. C32, C96, and C288: uninoculated plants grown at 32, 96, and 288 µM of phosphate, respectively. M32, M96, and M288: plants inoculated with *F. mosseae* and grown at 32, 96, and 288 µM of phosphate, respectively. Different letters indicate significant differences between the various treatments. Moreover, a two-way ANOVA is presented in the inset of each graph and considers the two factors phosphatic nutrition (P) and fungus (F) and their interaction (F * P). ns not significant; * *p* < 0.05; ** *p* < 0.01; *** *p* < 0.0001.

In C plants, the artemisinin concentration in leaves (Figure 3) proportionally decreased when P concentration increased in the substrate, with significant differences within all the C treatments. The P factor was responsible for this observed trend (two-way ANOVA). C32 and C96 plants presented the highest artemisinin abundance. These two plant treatments were significantly different from all the others. Irrespective of the P level, no significant differences were recorded in M plants, although a little decrease in artemisinin concentration was observed in M96 plants. Inoculated plants were quite similar to controls grown at 288 µM of phosphate. The fungus factor (F) was the main variable determining a low artemisinin concentration in the inoculated plants, as underlined by the two-way ANOVA.

### 3.5. Nutrient Concentration in the Different Plant Organs

Regarding the nutrient uptake (Table 3) in the different plant organs, the phosphorus concentration was proportional to its availability in the substrate, both in shoot and in root, regardless of the fungal inoculation. This strong effect of P nutrition was also confirmed by the two-way ANOVA. Although no significant differences were observed between C and M plants grown at the same P level, the inoculated ones showed, in general, an increased concentration of phosphorus in both organs. Moreover, the phosphorus concentration was similar in the shoot and the root of each treatment, whereas M32 plants displayed a phosphorus content in root 1.4 time higher than that recorded in shoot.

The uptake of K and S (Table 3) did not show any variation between the different treatments, regardless of the plant organs.

The magnesium (Mg) concentration (Table 3) was higher in the root of C32 and C96 plants (if compared to the other treatments), while lower values were detected in all ino-culated plants. In this case, the two-way ANOVA highlighted the great influence of the fungus factor (F). No differences in Mg uptake were observed in the shoot.

The sodium (Na) (Table 3) concentration in the shoot increased proportionally to the P level in the substrate both in control and inoculated plants. Confirmed by the two-way ANOVA, phosphatic nutrition (P factor) was the only factor responsible for this result. Instead, an opposite trend in Na root concentration was observed: C96 and C288 plants showed the highest values, whereas inoculated ones presented a proportional increase in Na as the P concentration ranged from 32 to 288 µM. In addition, Na concentration in M plants was lower than that measured in C ones (Table 3). The fungus factor (F) significantly affected this parameter (two-way ANOVA).

### 3.6. Principal Component Analysis—PCA

**Figure 4:**
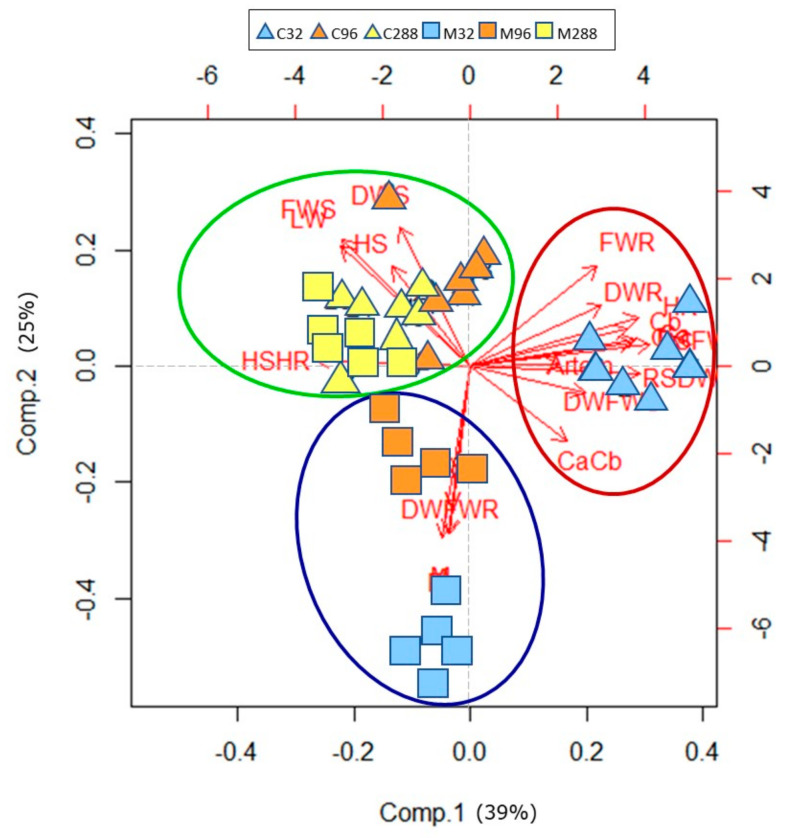
PCA of all plant growth parameters, photosynthetic pigment, and artemisinin concentration of *A. annua* plants grown at three different P levels (32, 96, and 288 μM) and inoculated (M) or not (C) with *F. mosseae* BEG 12. Plants of each group are indicated by different geometrical colored forms: C32 (light-blue triangle), C96 (orange triangle), C288 (yellow triangle), M32 (light-blue square), M96 (orange square), and M288 (yellow square). The three colored circles identify the groups described in the text (red: group 1; blue: group 2; green: group 3).

The PCA showed that the components 1 and 2 explained 39% and 25% of the total variance, respectively (Figure 4).

The different plant treatments are clustered into three distinct groups (green, blue, and red circles), each group clustered alone. The first group (red in Figure 4) is composed of control plants grown at 32 µM of phosphate (light-blue triangles) and is positively related with a set of parameters, such as leaf photosynthetic pigment and artemisinin concentrations, root fresh and dry weights (besides their ratio), and root length. The second group (blue circle in Figure 4) is represented by M plants grown at 32 µM and 96 µM of phosphate (light-blue and orange squares, respectively). This cluster is positioned along the negative branch of the ordinate axis (component 2). These plants are positively correlated with mycorrhizal parameters (F%, M%, and A%) and with the root DW/FW ratio. The third group (green circle in Figure 4) includes the plants grown at the highest P level inoculated (M288—yellow squares) or not (C288—yellow squares) and those uninoculated grown at 96 µM of phosphate (orange triangles). All these treatments are positively correlated with the shoot biomass parameters (shoot fresh and dry weights, stem height, and leaf weights).

## 4. Discussion

### 4.1. Mycorrhization and Plant Growth Parameters

The microbial community of the rhizosphere is complex, and the microbial plant-growth-promoting activity often occurs by synergistic interactions between diverse microbial taxa, as reported in previous studies [26,27,31,33]. In this work, we focused our attention on the effects of one fungal isolate on *A. annua* plants; for this reason, a sterilized substrate was used. The growth and physiology of *A. annua* plants were differently influenced both by mycorrhizal inoculation and by P nutrition. The establishment of the mycorrhizal symbiosis was negatively correlated with the increasing P concentration in the nutrient solution. Consistently, the highest colonization was observed in M32 plants, whereas those grown at the highest P concentration (M288) showed the lowest values. These latter plants did not show any trace of arbuscules, suggesting limited exchanges between the two partners. Many studies report that a high P concentration in the culture medium inhibits the ability of the fungus to establish an efficient symbiosis with plant roots [72,73,74]. Moreover, as confirmed by the PCA, M32 and M96 plants (Figure 4, blue circle) were positively associated to mycorrhizal parameters and were well separated by the M288 plants (that clustered with C288 ones; Figure 4, green circle).

In this work, a positive correlation between plant growth parameters and P nutrition was observed.

The shoot and root biomass production showed different trends in relation to either the P nutrition or mycorrhization. The aerial biomass increased proportionally to pho-sphate availability, according also to the PCA, in which C288 and C96 plants clustered together (Figure 4, green circle) and were positively correlated to the shoot biomass parameters. It is well known that a high P concentration in soil/medium can improve yield and aerial biomass production in different plant species [21,23,43,64]. Plants grown at intermediate P concentrations showed a different trend in response to the absence/presence of the fungus. Within controls, plants grown at 96 µM of phosphate reached the maximum values of shoot and leaf biomass, as displayed by the C288 plants (that in addition were quite similar to M288), while among the M plants, a significant reduction in this parameter was recorded in M96 plants if compared to those grown at 288 µM of phosphate. As previously observed [22,60,75], the reduction in the shoot and leaf weight could be ascribed to the fungal colonization. However, other studies have shown a higher biomass production in mycorrhizal plants grown at low P levels if compared to uninoculated ones [21,27,76]. Moreover, mycorrhizal inoculation affected plant height, suggesting that the fungus could influence not only the root growth [77,78] but also the epigeous biomass architecture [31,48,79], probably due to a modulation of the hormonal biosynthetic pathways [80,81]. The root biomass production showed a different trend compared to that observed for the shoot: a reduction was recorded in C plants at an increasing P concentration in the substrate. In contrast, the mycorrhizal plants presented similar values, independently of P nutrition and colonization level. This phenomenon was previously reported in many studies and seems to be due to the plant root ability to increase the nu-trient absorption surface by modifying the root system architecture [78,82,83]. In fact, in uninoculated plants, the P level played a key role in the root development. The root growth was probably boosted when phosphate was provided at low (32 µM) or intermediate (96 µM) concentrations, while this effect was not evident at the highest P level (288 µM). As confirmed by PCA, C32 plants positively correlated with root biomass parameters (Figure 4, red circle), while M32 plants, having the highest values of colonization, showed a small root growth, probably due to the exchanged nutrients between plant and fungus [32,43,84].

The ratio between dry and fresh weights of shoot decreased when the P concentration in the nutrient solution increased, underlining a better hydration state in plants grown at 288 µM of phosphate, independently of the inoculation. The improvement in mineral nutrition was involved in the amelioration of the water status, as reported also by Waraich and coworkers [85]. The same trend was observed for root dry/fresh weight in M plants, while no differences were recorded between C plants. These data are in contrast to those reported in the literature that underlined an improvement in water content in mycorrhizal plants [48,86]. The root/shoot ratio is one of the parameters that are modulated by P level and mycorrhization, being a good index of soil fertility and plant health [87]. More in detail, an increase in the root/shoot ratio occurred in uninoculated plants when the P concentration decreased in the growth medium [63,64], consistently with a great mineral mobilization toward the root [22,64,88,89]. According to these observations, our data showed a high value of the root/shoot ratio in C plants grown at the lowest P concentration, while a different trend was recorded between the M plants, which did not differ between the various treatments. As reported by Smith and Read [48], and also in our case, the low P input (32 µM) in the substrate promoted symbiosis and resulted in a decrease in the root/shoot ratio in M plants compared to C ones. Although M32 plants presented values quite similar to those presented by M96 and M288, the latter two showed a significant reduction in AM colonization, making them comparable either to C96 or to C288 plants. Therefore, we suppose that in this case, the increase in the P level was responsible for the root/shoot ratio decrease.

### 4.2. Photosynthetic Pigments

The plants grown at the lowest P level showed the highest concentrations of Chla, Chlb, and carotenoids, if compared to all the other treatments, independently of the ino-culation status. These data agree with the work of Rao and Terry [90], in which a high content of chlorophylls in plants grown at low phosphate availability was observed. This could be related to a mechanism involved in the prevention of photoinhibition under phosphorus deficiency, leading to an alteration in the thylakoid membrane composition and to a reduction in phosphorus demand for membranes, making it available for the photosynthesis [64,91]. Moreover, in other studies performed on *A. annua* plants, no differences between controls and M plants were recorded for the chlorophyll concentration [21,28]. Although an increase in antioxidant molecules in mycorrhizal plants has been reported in the literature [28,92,93], our data showed an increased concentration of carotenoids only in C32 plants, underlining that these plants were probably stressed due to the low availability of nutrient. However, this phosphorus deficit was restored by AM inoculation in M32 plants, leading to a reduction in carotenoids, also displayed in plants grown at higher P levels.

### 4.3. Artemisinin Production

The artemisinin concentration in the leaves was inversely proportional to the P concentration in the substrate. This result differed from those reported in other studies on *A. annua* plants grown in soil and fertilized with a P concentration comparable with our intermediate and highest levels [11,21]. The fungus effects were limited. In fact, in the ino-culated plants, a correlation to the P concentration was not observed: the artemisinin amount did not change at the three P levels. This result is also in contrast with most of the studies reported in the literature where an increase in the artemisinin content in mycorrhizal plants was recorded [21,22,28,31,94]. However, it is well known that the effects of distinct fungal species could differently modulate the secondary metabolite production [35,93,95,96]. Consistently, in another study [24], the same fungus (*F. mosseae*) used in our experiment did not enhance the artemisinin content, and in other plants, a neutral effect of the inoculation on the secondary metabolite production was reported [60]. Moreover, in previous works, a direct correlation between the chlorophyll and the artemisinin concentration has been found [1,97]. A similar trend was observed in our study except for C96 plants. Alternatively, since the last reaction of the artemisinin biosynthetic pathway is not enzymatic but is a photo- or a self-oxidation reaction [17] and in many cases, the secondary metabolite production is associated with reactive oxygen species (ROS) produced by stress [98], the higher artemisinin amount measured in plants grown at the lo-west P concentration could be related to the phosphorus starvation. Therefore, in our case, a better uptake of phosphate was not correlated with an increase in artemisinin production and the inoculation with *F. mosseae* did not enhance the plant’s secondary metabolism.

### 4.4. Nutrient Uptake

The lowest concentration of P used in our experimental system has already been tested by our research group on various crops having different nutritional needs, often in the presence of soil beneficial microorganisms, showing an improvement in growth, production, and yield. As mentioned before, *A. annua* is a ruderal plant, which does not require a large supply of nutrients. Therefore, in the present study, this P level (32 µM) was enough either for the plant growth and development or for the establishment of AM symbiosis. However, the expected growth effect due to inoculation was not observed, probably since the plant was not exclusively dependent on the fungus for P nutrition. Consi-stently with what we expected and with the literature [99,100], phosphorus uptake increased with the enhancement of P supply in the substrate both in control and inoculated plants, according to the two-way ANOVA, where P was the only factor responsible for this trend. In contrast with data reported in the literature indicating that mycorrhizal inoculation improves the phosphorus uptake, making it available for the plant in soluble forms [43,74,84,101,102,103,104,105], in the present work, the fungus did not significantly affect the phosphorus uptake. However, in our study, phosphorus was already added in a soluble form as a nutrient solution ready for plant uptake.

Variations in the Mg uptake were observed only in the root, in accordance with stu-dies reporting a decreased concentration of Mg at high P levels [106]. The AM fungus reduced the Mg concentration, and this is in contrast with works in which AM inoculation enhanced this uptake [102]. Mg plays an important role in chlorophyll production and thus in photosynthesis [64]. In fact, in our work, a lower chlorophyll concentration in ino-culated plants was observed.

The fungus also reduced the Na concentration in the root, whereas in the control plants, the Na concentrations were higher both at 96 and 288 µM of phosphate. This observation confirmed that AM fungi strongly lowered the Na accumulation [107]. Instead, in the shoot, only the phosphate factor influenced the Na concentration, which increased proportionally to the P level in the substrate. This is probably due to the major concentration of Na in the substrate, because in the Long Ashton nutrient solution, the phosphorus was in the form of NaH_2_PO_4_.

## 5. Conclusions

This work highlighted the importance of selecting the right fungus/plant combination, because the involved AM fungus defines the symbiosis functioning and the effect on plant growth and metabolism. The compatibility between the two symbionts is a crucial factor that may determine the effectiveness of the fungus and the responsiveness of the plants to the symbiosis. In fact, despite the ubiquity of the AM symbiosis, plant growth responses to AM fungi vary widely along a continuum from positive to negative and can be influenced by both plant and fungal species involved in the symbiosis, soil nutrient availability, and growth conditions. All these factors could act as agents of selection on the symbiosis. Another point to be considered is that the rhizosphere microbial community is complex and the microbial plant-growth-promoting activity often occurs by synergistic interactions between diverse microbial taxa. Further investigations will be necessary to assess the effect of different bioformulations containing a mix of AM fungi (together also with plant-growth-promoting bacteria) to gain the optimal results in terms of plant biomass and artemisinin production.

## Figures and Tables

**Table 1:** Summary of the different plant treatments included in the experimental design. C32, C96, and C288: uninoculated plants (7 plants per treatment) grown at 32, 96, and 288 µM of P, respe-ctively. M32, M96, and M288: plants inoculated with *F. mosseae* (5 plants per treatment) and grown with 32, 96, and 288 µM of P, respectively.

Fungal Treatment	P Concentration in the LA Solution	Plant Treatment Abbreviation
No fungus	32 µM	C32
No fungus	96 µM	C96
No fungus	288 µM	C288
*Funneliformis mosseae* BEG12	32 µM	M32
*Funneliformis mosseae* BEG12	96 µM	M96
*Funneliformis mosseae* BEG12	288 µM	M288

**Table 2:** Plant growth parameters. The different growth parameters recorded in *A. annua* plants are reported: shoot FW and DW (g), shoot DW/FW ratio, stem height (cm), leaf FW and DW (g), root FW and DW (g), root DW/FW ratio, root length (cm), root/shoot FW ratio, root/shoot DW ratio, and stem height/root length ratio. C32, C96, and C288: uninoculated plants grown at 32, 96, and 288 µM of phosphate, respectively; M32, M96, and M288: plants inoculated with *F. mosseae* and grown at 32, 96, and 288 µM of phosphate, respectively. Data (mean ± standard error) were analyzed by a one-way ANOVA with a Fisher post-hoc test. Different letters within each row indicate significant differences between the treatments (*p* < 0.05). The column on the right shows data obtained by the two-way ANOVA and considers the two factors phosphatic nutrition (P) and fungus (F) and their interaction (F * P); ns: not significant; * *p* < 0.05; ** *p* < 0.01; *** *p* < 0.0001.

	Plant Treatments						Two-Way ANOVA		
Parameters	C32	C96	C288	M32	M96	M288	P	F	F * P
**Shoot FW (g)**	7.063 ± 0.339 **c**	12.489 ± 0.675 **a**	12.647 ± 0.449 **a**	6.723 ± 0.606 **c**	8.900 ± 0.565 **b**	12.587 ± 0.081 **a**	***	*	*
**Shoot DW (g)**	1.441 ± 0.063 **cd**	2.100 ± 0.093 **a**	1.877 ± 0.124 **ab**	1.213 ± 0.159 **d**	1.503 ± 0.235 **bcd**	1.687 ± 0.054 **bc**	**	*	ns
**Shoot DW/FW**	0.205 ± 0.006 **a**	0.169 ± 0.006 **b**	0.148 ± 0.006 **cd**	0.148 ± 0.007 **b**	0.168 ± 0.020 **bc**	0.134 ± 0.004 **d**	***	ns	ns
**Stem height (cm)**	14.571 ± 0.369 **c**	20.214 ± 1.430 **a**	17.914 ± 0.582 **ab**	15.100 ± 0.551 **bc**	14.833 ± 2.987 **bc**	15.100 ± 0.208 **bc**	ns	*	ns
**Leaf FW (g)**	4.531 ± 0.231 **c**	8.464 ± 0.467 **a**	8.463 ± 0.538 **a**	4.447 ± 0.401 **bc**	6.147 ± 0.471 **b**	9.237 ± 0.052 **a**	***	ns	ns
**Leaf DW (g)**	0.946 ± 0.052 **b**	1.400 ± 0.078 **a**	1.239 ± 0.100 **a**	0.850 ± 0.122 **b**	1.163 ± 0.16 **ab**	1.293 ± 0.003 **a**	**	ns	ns
**Root FW (g)**	7.949 ± 0.694 **a**	7.090 ± 0.892 **a**	4.189 ± 0.525 **b**	3.103 ± 0.820 **b**	3.843 ± 0.334 **b**	4.217 ± 0.105 **b**	ns	***	*
**Root DW (g)**	0.787 ± 0.085 **a**	0.660 ± 0.077 **ab**	0.414 ± 0.047 **c**	0.423 ± 0.128 **bc**	0.473 ± 0.068 **bc**	0.410 ± 0.097 **bc**	ns	*	ns
**Root DW/FW (g)**	0.099 ± 0.004 **bc**	0.095 ± 0.005 **c**	0.100 ± 0.001 **bc**	0.134 ± 0.005 **a**	0.123 ± 0.014 **ab**	0.096 ± 0.022 **bc**	ns	**	ns
**Root length (cm)**	51.857 ± 2.567 **a**	36.957 ± 2.652 **b**	24.671 ± 2.694 **c**	23.933 ± 1.126 **c**	27.767 ± 4.436 **bc**	26.200 ± 2.914 **c**	**	***	***
**Root/shoot FW**	1.124 ± 0.082 **a**	0.563 ± 0.061 **b**	0.329 ± 0.038 **c**	0.450 ± 0.081 **bc**	0.433 ± 0.020 **bc**	0.333 ± 0.009 **c**	***	***	***
**Root/shoot DW**	0.544 ± 0.048 **a**	0.311 ± 0.033 **b**	0.217 ± 0.021 **b**	0.333 ± 0.060 **b**	0.317 ± 0.032 **b**	0.240 ± 0.055 **b**	***	ns	*
**Stem height/root length**	0.286 ± 0.026 **c**	0.571 ± 0.061 **b**	0.771 ± 0.081 **a**	0.633 ± 0.033 **ab**	0.600 ± 0.200 **ab**	0.600 ± 0.058 **ab**	*	ns	*

**Table 3:** Element concentrations (mg kg^−1^) in shoot and root. Data related to the concentrations of phosphorus, potassium, sulfur, magnesium, and sodium in the shoot and root of *A. annua* plants. C32, C96, and C288 specify the uninoculated plants grown at 32, 96, and 288 μM of phosphate, respectively; M32, M96, and M288 specify the plants inoculated with the fungus *F. mosseae* BEG12 grown at 32, 96, and 288 μM of phosphate, respectively. Data (mean ± standard error) were analyzed by a one-way ANOVA with a Fisher post-hoc test. Different letters within each row indicate significant differences among the treatments (*p* < 0.05). The last three columns show data obtained by a two-way ANOVA and considers the two factors phosphatic nutrition (P) and fungus (F) and their interaction (F * P). ns not significant; * *p* < 0.05; ** *p* < 0.01; *** *p* < 0.0001.

	Plant Treatments						Two-Way ANOVA		
Shoot	C32	C96	C288	M32	M96	M288	P	F	F * P
P (mg Kg^−1^)	221.124 ± 20.662 **c**	339.754 ± 55.78 **bc**	779.161 ± 82.347 **a**	293.395 ± 53.127 **bc**	415.841 ± 78.893 **b**	839.561 ± 62.995 **a**	***	ns	ns
K (mg Kg^−1^)	14,803.52 ± 967.717 **a**	16,564.5 ± 1917.294 **a**	18,652.62 ± 1038.739 **a**	15,342.78 ± 798.021 **a**	18,258.80 ± 1189.825 **a**	21,823.20 ± 1997.428 **a**	ns	ns	ns
S (mg Kg^−1^)	846.989 ± 85.015 **a**	767.614 ± 91.817 **a**	717.273 ± 35.069 **a**	640.817 ± 54.926 **a**	788.680 ± 59.105 **a**	924.677 ± 64.822 **a**	ns	ns	ns
Mg (mg Kg^−1^)	1035.383 ± 100.02 **a**	15,342.78 ± 798.021 **a**	640.817 ± 54.926 **a**	853.243 ± 168.847 **a**	1019.653 ± 172.100 **a**	1235.59 ± 308.338 **a**	ns	ns	ns
Na (mg Kg^−1^)	133.679 ± 16.216 **b**	153.279 ± 12.011 **b**	247.156 ± 52.729 **a**c	143.207 ± 18.522 **bc**	233.837 ± 50.435 **ab**	293.107 ± 40.678 **a**	**	ns	ns
**Root**	**C32**	**C96**	**C288**	**M32**	**M96**	**M288**			
P (mg Kg^−1^)	275.757 ± 20.662 **b**	414.767 ± 55.78 **b**	744.550 ± 82.347 **a**	416.122 ± 53.127 **b**	444.173 ± 78.893 **b**	863.473 ± 62.995 **a**	***	ns	ns
K (mg Kg^−1^)	17,357.52 ± 1110.445 **a**	12,532.82 ± 1671.586 **a**	13,450.90 ± 1754.138 **a**	14,105.12 ± 396.816 **a**	14,160.66 ± 1561.905 **a**	1226.813 ± 243.734 **a**	ns	ns	ns
S (mg Kg^−1^)	921.494 ± 88.324 **a**	1349.837 ± 125.182 **a**	1203.774 ± 132.662 **a**	897.983 ± 90.698 **a**	829.893 ± 115.171 **a**	1226.813 ± 243.734 **a**	ns	ns	ns
Mg (mg Kg^−1^)	1801.303 ± 293.776 **ab**	2288.069 ± 366.821 **a**	1190.891 ± 171.064 **bc**	797.967 ± 6.885 **c**	1032.543 ± 281.134 **bc**	1005.577 ± 262.912 **bc**	ns	**	ns
Na (mg Kg^−1^)	1005.599 ± 132.433 **bc**	2368.611 ± 456.079 **a**	1754.737 ± 317.798 **ab**	351.343 ± 75.64 **c**	629.317 ± 109.468 **c**	1339.047 ± 106.568 **abc**	ns	*	ns

## Data Availability

The data presented in this study are available on request from the corresponding author.

## Supplementary Materials

The following are available online at https://www.mdpi.com/article/10.3390/life12040497/s1: Figure S1: Microscopic view of *A. annua* root colonization by *F. mosseae*; Figure S2: Artemisinin HPLC chromatogram (A) and MS spectrum (B).
