# Supplementary material for: Impact of Phosphatic Nutrition on Growth Parameters and Artemisinin Production in Artemisia annua Plants Inoculated or Not with Funneliformis mosseae
Source: Life (Basel). 2022 Mar 29;12(4):497. doi: 10.3390/life12040497 (PMC9025405; doi:10.3390/life12040497)
Supplement: Supplementary file 1 [file life-12-00497-s001.zip › life-1655407-supplementary.pdf]

life

Article

Impact of Phosphatic Nutrition on Growth Parameters and Artemisinin Production in *Artemisia annua* Plants Inoculated or Not with *Funneliformis mosseae*

Valeria Todeschini [1,*], Flavio Anastasia [1], Nadia Massa [1], Francesco Marsano [1], Patrizia Cesaro [1], Elisa Bona [2], Elisa Gamalero [1], Ludovica Oddi [3] and Guido Lingua [1]

1 Dipartimento di Scienze ed Innovazione Tecnologica, Università del Piemonte Orientale, 15121, Alessandria, Italy; flavio.anastasia@uniupo.it (F.A.); nadia.massa@uniupo.it (N.M.); francesco.marsano@uniupo.it (F.M.); patrizia.cesaro@uniupo.it (P.C.) elisa.gamalero@uniupo.it (E.G.); guido.lingua@uniupo.it (G.L.)
2 Dipartimento per lo Sviluppo Sostenibile e la Transizione Ecologica, Università del Piemonte Orientale, 13100, Vercelli, Italy; elisa.bona@uniupo.it
3 Dipartimento di Scienze della Vita e Biologia dei Sistemi, Università degli studi di Torino, 10123, Torino, Italy; ludovica.oddi@unito.it
* Correspondence: valeria.todeschini@uniupo.it; Tel.: +39-0131-360210

Supplementary

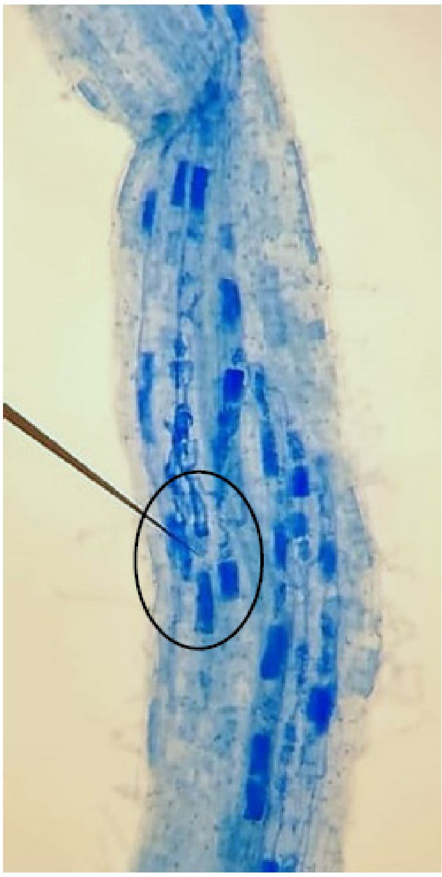

Figure S1. Microscopic view of *A. annua* root colonization by *F. mosseae*. Part of 1cm-long root piece from root of M32 plant stained with lactic blue. Fungal hyphae and arbuscules are indicated by the black circle. Magnification 10x.

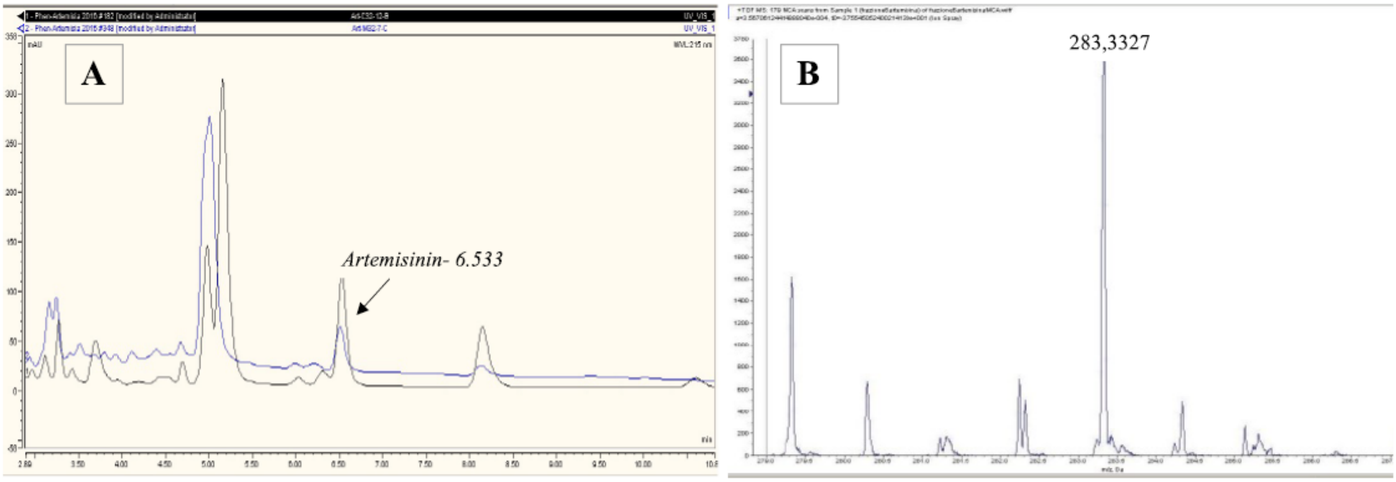

Figure S2. Artemisinin HPLC chromatogram and MS spectrum. Example of artemisinin HPLC chromatogram obtained by leaves of *A. annua* plants inoculated (blue) or not (grey) grown at 32µM of P (A). MS spectrum of protonated artemisinin obtained by MALDI-TOF analysis Voyager DE-PRO (B).
